# The effect of temperature and breathing pattern on the surface activity of ground squirrel pulmonary surfactant

**DOI:** 10.1242/jeb.249280

**Published:** 2024-10-08

**Authors:** Akash Tejura, Mengxi Sun, Lynda McCaig, James Staples, Ruud Veldhuizen

**Affiliations:** ^1^ Departments of Physiology and Pharmacology, Medicine, Western University, London, ON, Canada, N6A 5C1; ^2^ Department of Biology, Western University, London, ON, Canada, N6A 3K7

**Keywords:** Surface film, Hibernation, Surface tension

## Abstract

This study investigates how hibernation affects the surface activity of pulmonary surfactant with respect to temperature and breathing pattern. Surfactant was isolated from a hibernating species, the 13-lined ground squirrel, and a homeotherm, the rabbit, and analysed for biophysical properties on a constrained sessile drop surfactometer. The results showed that surfactant from ground squirrels reduced surface tension better at low temperatures, including when mimicking episodic breathing, as compared with rabbit surfactant. In addition, low temperature adaptation was also observed using only the hydrophobic components of surfactant from ground squirrels. Overall, the data support the conclusion that ground squirrel surfactant has adapted to maintain surface activity during low temperature episodic breathing patterns, and that temperature adaptation is maintained with the hydrophobic components of the surfactant.

## Teaser

**Summary:** Pulmonary surfactant from hibernating animals maintains the ability to reach low surface tensions during episodic breathing patterns and low temperatures. This activity is maintained within the hydrophobic component of surfactant.

## INTRODUCTION

During hibernation, body temperatures decrease to conserve energy during periods of cold ambient temperatures and associated food shortages. These low body temperatures are associated with corresponding decreases in metabolic rate, heart rate and ventilation (Sprenger and Milsom, 2022; Staples, 2016). Focusing on ventilation, the breathing rate during hibernation has been measured as low as 3 breaths min^−1^ (Sprenger and Milsom, 2022). Furthermore, the breathing pattern consists of rapid episodic breaths, or gasps, followed by periods of apnoea. Our research goal is to understand the impact of hibernation on the mechanics associated with inflation and deflation of the lung, with a specific focus on pulmonary surfactant.

Pulmonary surfactant lines the alveolar surface and is essential for normal ventilation (Goerke and Clements, 1985). Surfactant is composed of the hydrophobic components consisting of phospholipids, cholesterol and surfactant proteins B (SP-B) and SP-C, as well as hydrophilic components, most notably SP-A (Zuo et al., 2008). The biophysical activity of this material generates a phospholipid-rich surface film that reduces the surface tension to allow for inflation of the lung with relative ease (Goerke and Clements, 1985). A large number of studies have explored the mechanisms by which surfactant performs this function (Bakshi et al., 2008; Gomez-Gil et al., 2009; Pérez-Gil, 2008; Possmayer et al., 2023; Schief et al., 2003). Although specific details vary, most studies agree that surfactant needs to be sufficiently fluid to form and maintain a surface film, yet be sufficiently stiff and stable to reduce the surface tension to near zero values upon compression (exhalation) to stabilize the lung. However, the fluidity and stiffness of phospholipid films depend greatly on temperature, raising the question of how this material functions at the low body temperatures encountered by hibernators, such as the 13-lined ground squirrel (Suri et al., 2012, 2013).

Earlier research explored the hypothesis that the lipid composition of surfactant, specifically the surface-active large aggregate subfraction, would change during the hibernating period (Suri et al., 2012, 2013). It was predicted that during hibernation, surfactant would contain more unsaturated phospholipids and more cholesterol, which would increase surfactant fluidity and allow for proper function at low temperatures. The data, specifically as they relate to cholesterol, are inconclusive, with evidence of both altered and unaltered levels of cholesterol associated with low body temperatures depending on the species (Codd et al., 2000, 2002; Possmayer et al., 2023; Slocombe et al., 2000). Conversely, at 37°C, it was anticipated that surfactant would contain more solid phospholipids, such as the disaturated dipalmitoyl-phosphatidylcholine (DPPC). Although some notable exemptions exist, DPPC is the main phospholipid present in most mammalian surfactants and is crucial in the reduction of surface tension to low values in homeothermic animals (Lang et al., 2005; Possmayer et al., 2023). The data on the surfactant from hibernating 13-lined ground squirrel indicated an increase in some of the unsaturated phospholipids as compared with surfactant obtained during the summer-active period. Interestingly, however, surface activity measurements during dynamic compression–expansion cycles indicated that surfactant from both summer-active and hibernating squirrels performed equally well over a range of temperatures (Suri et al., 2012, 2013). Furthermore, ground squirrel surfactant was more functional at low temperatures than surfactant from pigs, regardless of whether it was obtained from hibernating of summer-active animals (Suri et al., 2012, 2013).

In the current study, we expand on the above observations by addressing two research questions. First, are the hydrophobic components of surfactant responsible for the temperature adaptation of ground squirrels? Second, is ground squirrel surfactant better adapted to reducing surface tension during episodic breathing patterns, as observed during hibernation?

## MATERIALS AND METHODS

### Surfactant preparations

Surfactant large aggregates were obtained from two adult, male, New Zealand white rabbits, *Oryctolagus cuniculus* (Linnaeus 1758) and approximately 10 adult 13-lined ground squirrels, *Ictidomys tridecemlineatus* (Mitchill 1821), of both sexes. These healthy animals were euthanized according to approved ethics protocols (Western University, animal care subcommittee protocols: #2018-054 and #012-016) for experimental studies unrelated to the current objectives. Briefly, a full lung lavage was performed by instilling saline into the lung of each animal until the lung was fully distended, the saline was withdrawn and reinstilled twice and this procedure was repeated an additional 2 times with fresh saline (Puligandla et al., 2000; Suri et al., 2013). The three lavages were combined, and large aggregates were obtained by centrifugation at 40,000 ***g*** for 15 min. The large aggregate pellets were resuspended in buffer (2.5 mmol l^−1^ Hepes, 0.15 mol l^−1^ NaCl, 1.5 mmol l^−1^ CaCl_2_, pH 7.4) and all samples from each species were combined. The concentration of surfactant in the pooled large aggregate samples was determined via a phospholipid–phosphorus assay as previously reported (Bligh and Dyer, 1959; Duck-Chong, 1979; Maruscak et al., 2008).

### Measurement of surface tension reduction

Functional assessment of the surfactants was performed on a constrained sessile drop surfactometer. Briefly, an 8.5 µl drop of each sample at 2.5 mg ml^−1^ in Hepes buffer (see above) was placed onto a pedestal within a temperature-controlled chamber set at 5, 24 or 37°C. A small pinhole within the pedestal was attached to a computer-controlled syringe containing water, allowing for accurate changes to the volume of the drop, thereby causing dynamic compression and expansion of the surface film (Graham et al., 2022; Zuo et al., 2004). Thus, by changing the drop volume, the syringe was used to mimic compression and expansion of the surfactant film as encountered *in vivo* during breathing. During the experiment, a camera (PixeLink PL-B771U) was utilized to take images of the surfactant sample, which allowed for the subsequent measurement of surface area and surface tension, based on the shape of the drop, using asymmetric drop-shape analysis (ADSA) software (Zuo et al., 2004).

### Experiment 1: assessing surfactant function of purified large aggregates at different temperatures

Surfactant large aggregates from both species were analysed on the constrained sessile drop surfactometer at the three different temperatures. Following placement of the surfactant on the pedestal, the sample was allowed to adsorb for 2 min followed by 20 dynamic compression–expansion cycles. These cycles were done at 1.5 s cycle^−1^ with an area compression of approximately 18–20% of the original surface area.

### Experiment 2: assessing surfactant function of extracted large aggregates at different temperatures

To explore the ability of the hydrophobic components of surfactant to account for the reduction of surface tension at different temperatures, large aggregate samples from both species underwent a chloroform extraction prior to analysis (Bligh and Dyer, 1959). The hydrophobic components (lipids, SP-B and SP-C) contained in the chloroform layer were isolated and dried under nitrogen. Following a phospholipid–phosphorus analysis (Duck-Chong, 1979), the extracted surfactants were resuspended in Hepes buffer at 2.5 mg ml^−1^ and were analysed as described above for experiment 1.

### Experiment 3: mimicking an episodic breathing pattern to assess surfactant function of large aggregates at different temperatures

To examine how the episodic breathing pattern of hibernating mammals (Sprenger and Milsom, 2022) affects surfactant function, we took advantage of the flexibility of the constrained sessile drop surfactometer. Similar to experiment 1, a drop of each of the samples was placed on the pedestal and left for 2 min to adsorb. Following this, the large gasps associated with hibernating breaths were simulated by expanding the drop such that the surface area was approximately 45% larger than its original surface area. This expansion was followed by a compression of the same magnitude followed by a subsequent expansion and compression of the same magnitude. This was followed by about 20–30 s of inactivity to simulate a period of apnoea, followed by two more large expansion–compressions. A second period of apnoea and a third set of expansion–compression cycles was performed to complete the assessment.

### Statistical analysis

Each experimental condition was tested 3 times on separate days; during each individual test, three technical replicates were performed and analysed. Statistical analysis was conducted using GraphPad Prism Software and included *t*-tests for comparisons between two measurements or two-way analysis of variance with a Tukey *post hoc* test for comparisons over multiple expansion–compression cycles. *P*-values <0.05 were considered significant.

## RESULTS AND DISCUSSION

### Experiment 1

**Fig. 1.**
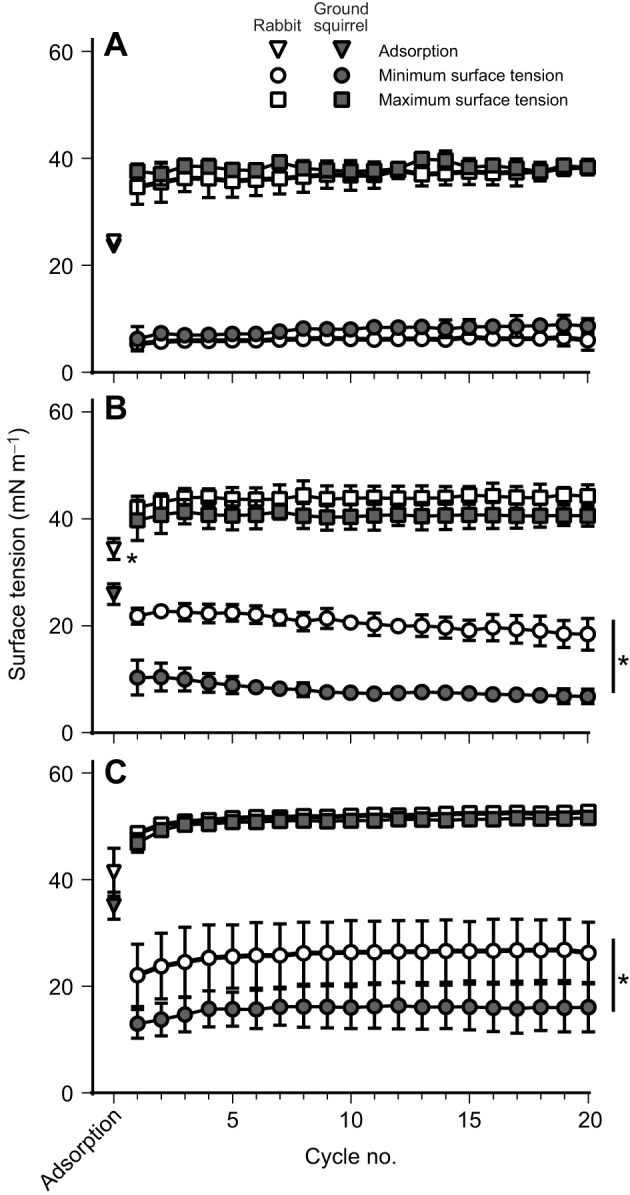
**Surface activity of isolated rabbit and ground squirrel pulmonary surfactant at different temperatures.** Surfactant samples were analysed on a constrained sessile drop surfactometer for adsorption, and minimum and maximum surface tension at (A) 37°C, (B) 22°C and (C) 5°C. *P<0.05 rabbit versus ground squirrel surfactant, using a *t*-test for adsorption values and two-way analysis of variance with a Tukey *post hoc* test for minimum and maximum surface tension values.

The first experiment examined the impact of temperature on the surface activity of rabbit and ground squirrel large aggregates. Shown in Fig. 1 are the surface tension after 2 min adsorption, minimum surface tension values upon dynamic compression, and maximum surface tension upon expansion. There were no significant differences among the surfactants at 37°C, with the values demonstrating values after adsorption near the equilibrium surface tension of 23 mN m^−1^, and minimum surface tension values below 5 mN m^−1^ (Fig. 1A). In contrast, at lower temperatures, ground squirrel surfactant had significantly lower surface tension values after adsorption and compression at 22°C (Fig. 1B), and significantly lower values of minimum surface tension at 5°C (Fig. 1C).

### Experiment 2

**Fig. 2.**
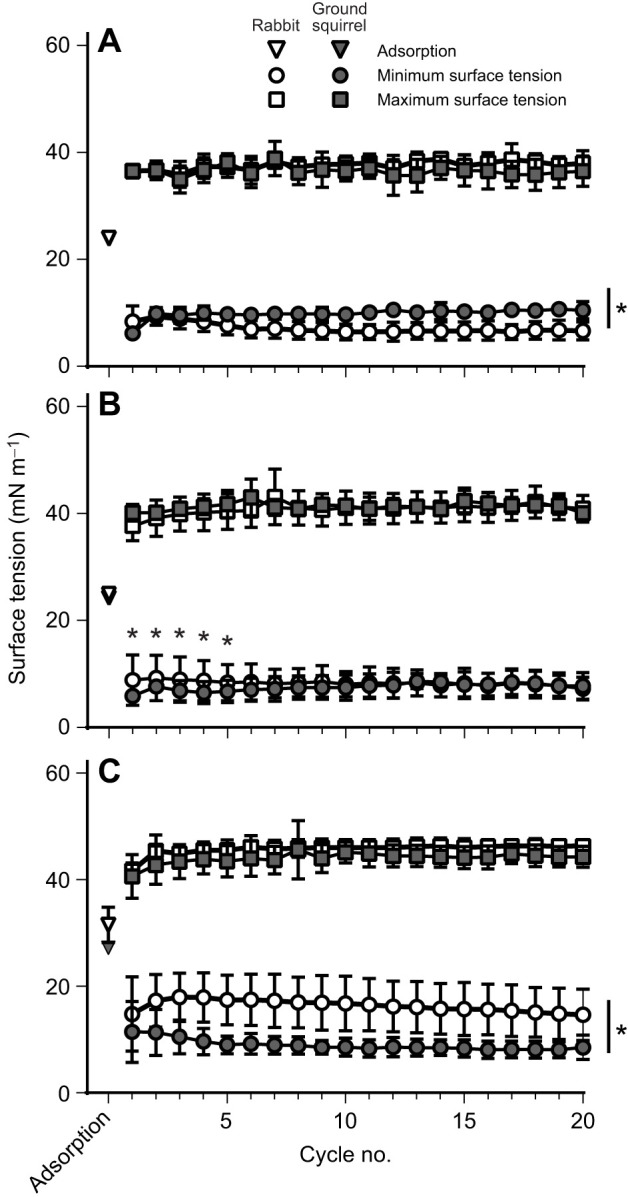
**Surface activity of extracted rabbit and ground squirrel surfactant at different temperatures.** Extracted surfactant samples were analysed on a constrained sessile drop surfactometer for adsorption, and minimum and maximum surface tension at (A) 37°C, (B) 22°C and (C) 5°C. *P<0.05 rabbit versus ground squirrel surfactant, using a *t*-test for adsorption values and two-way analysis of variance with a Tukey *post hoc* test for minimum and maximum surface tension values.

The results of the second experiment, in which resuspended chloroform extracts of the large aggregate samples were utilized, are shown in Fig. 2. Values obtained at 37°C (Fig. 2A) indicate that extracted surfactant from the two species had similar adsorption and maximum surface tension values. Minimum surface tension was slightly, but significantly, higher in the samples from ground squirrels as compared with those from rabbits. At 22°C (Fig. 2B), the samples from the two species behaved similarly except for minimum surface tension values during the first five compression–expansion cycles, which revealed higher values for the rabbit surfactant. Finally, at 5°C (Fig. 2C), the minimum surface tension values were higher in samples from rabbits as compared with ground squirrels, with no statistically significant differences in adsorption or maximum surface tension.

### Experiment 3

**Fig. 3.**
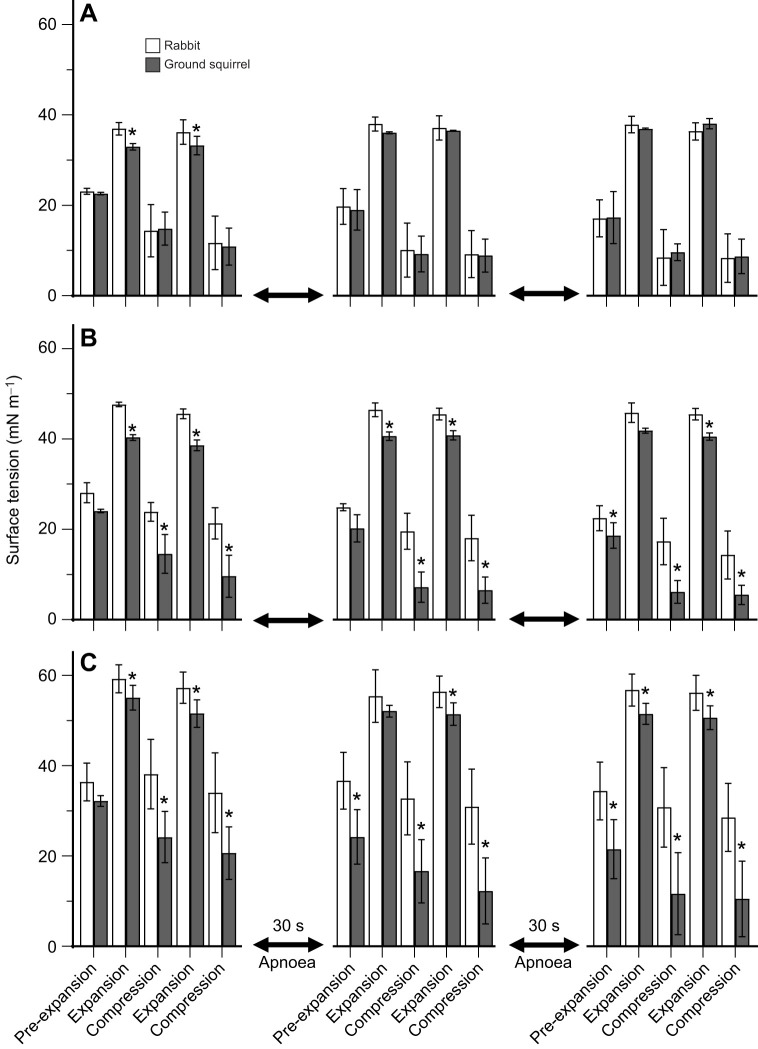
**Surface activity of isolated rabbit and ground squirrel surfactant at different temperatures using an episodic expansion–compression pattern.** Surfactant samples were analysed on a constrained sessile drop surfactometer and surface tension was determined at different points along the episodic pattern at (A) 37°C, (B) 22°C and (C) 5°C. *P<0.05 rabbit versus ground squirrel surfactant, using a two-way analysis of variance with a Tukey *post hoc* test.

To evaluate the impact of the breathing pattern observed during hibernation, the results depicted in Fig. 3 show surface tensions observed during three sets of two large expansion–compression cycles separated by a period of apnoea. The results at 37°C (Fig. 3A) show similar surface tension values for the two species throughout the experiment, except for slightly lower values during the first two expansion cycles with ground squirrel surfactant as compared with the rabbit material. Compared with rabbit surfactant, lower surface tension values were observed with ground squirrel surfactant at 22°C and 37°C throughout the experiments, reaching statistical significance for most of the data points (Fig. 3B,C).

### Analysis of results

Previous literature has provided evidence that surfactant obtained from hibernating mammals is more effective at reducing surface tension across various temperatures than that of homeothermic mammals (Suri et al., 2012, 2013). In the current experiments, we initially confirmed these observations and built on them by addressing two specific aspects of the surfactant function. First, we evaluated the role of the hydrophobic components of surfactant in the temperature adaptation of ground squirrels. Our results indicated that extracted surfactant from ground squirrels was better at reducing surface tension at low temperatures than extracted rabbit surfactant, leading to the conclusion that, in part, temperature adaptation was maintained within the hydrophobic components of ground squirrel surfactant. The second aspect we explored was the impact that the episodic breathing patterns during hibernation may have on surfactant function. Our results indicated lower surface tension with samples from ground squirrels when analysed over several large expansion–compression cycles, intermixed with periods of apnoea. These data support the conclusion that ground squirrel surfactant is adapted to maintain functionality under hibernation conditions.

Whereas the above conclusions are based on the direct comparison of ground squirrels and rabbit surfactant within each experiment, it is also valuable to compare the different conditions. Specifically, comparisons between the extracted and non-extracted samples may provide information on the impact of the different components of surfactant. There is extensive literature from homeothermic mammals that indicates that surface tension reduction by surfactant is mainly associated with its hydrophobic components, the lipids, SP-B and SP-C (Almlén et al., 2008; Possmayer et al., 2023; Whitsett and Weaver, 2002). These hydrophobic components were present in our extracted samples with the hydrophilic components, including SP-A, being removed during the extraction process. SP-A is not essential for function but has been reported to enhance surfactant function under stress conditions such as at low concentrations or when inhibitors are present (Cockshutt et al., 1990; Ikegami et al., 1998; Possmayer et al., 2023). The comparisons of our experiments with unextracted versus extracted surfactant revealed that, at low temperatures, the difference in minimum surface tensions between the two species was much larger with the unextracted material. Furthermore, it appeared that extracted rabbit surfactant functioned better at low temperatures than unextracted surfactant. The tentative interpretation of these observations is that SP-A does not influence surfactant activity at low temperatures in ground squirrels, but may in fact become inhibitory in rabbits at low temperatures. However, this requires further analysis, possibly after the isolation and comparison of rabbit and ground squirrel SP-A. Furthermore, the impact of other hydrophilic components, removed during the extraction process, cannot be excluded.

Methodologically, our experimental approach utilized the constrained sessile drop surfactometer in which the volume of a drop of surfactant is altered via a computer-controlled syringe within an environmental control chamber. This experimental set-up offers excellent control of various experimental conditions, including exposure to aerosols, humidity and imaging of the droplet, among others (Possmayer et al., 2023; Valle et al., 2015; Zhang et al., 2012; Zuo et al., 2005). Our experiment took advantage of this experimental flexibility by not only examining temperature but also mimicking the gasping breaths that hibernating animals utilize. The results of our experiments with this episodic breathing pattern not only support the general concept that ground squirrel surfactant has improved surface activity at lower temperatures but also provide new information indicating ground squirrel surfactant maintained lower surface activity during irregular expansion–compression cycling involving rapid expansion followed by simulated periods of apnoea.

The limitations of our study include the use of pooled surfactant samples from multiple animals to obtain sufficient quantities of surfactant to perform all experimental conditions. This did not allow us to assess biological variability or differences between males and females. It should be noted however that, under our control conditions (Fig. 1), these pooled samples behaved as expected from the literature (Suri et al., 2012, 2013). Another limitation is that in our experiment mimicking the hibernating breathing pattern, the 30 s apnoea period and 45% surface area increase during expansion only reflect the general concept of what occurs *in vivo* rather than specific values for these variables.

In conclusion, this study demonstrates that the hydrophobic component of surfactant from 13-lined ground squirrels is capable of maintaining functional surfactant at a wide temperature range. In addition, ground squirrel surfactant has adapted to maintain surface activity during episodic breathing patterns.

## References

[JEB249280C1] Almlén, A., Stichtenoth, G., Linderholm, B., Haegerstrand-Björkman, M., Robertson, B., Johansson, J. and Curstedt, T. (2008). Surfactant proteins B and C are both necessary for alveolar stability at end expiration in premature rabbits with respiratory distress syndrome. *J. Appl. Physiol. (1985)* 104, 1101-1108. 10.1152/japplphysiol.00865.2007 18276900

[JEB249280C2] Bakshi, M. S., Zhao, L., Smith, R., Possmayer, F. and Petersen, N. O. (2008). Metal nanoparticle pollutants interfere with pulmonary surfactant function *in vitro*. *Biophys. J.* 94, 855-868. 10.1529/biophysj.107.106971 17890383 PMC2186259

[JEB249280C3] Bligh, E. G. and Dyer, W. J. (1959). A rapid method of total lipid extraction and purification. *Can. J. Biochem. Physiol.* 37, 911-917. 10.1139/y59-099 13671378

[JEB249280C4] Cockshutt, A. M., Weitz, J. and Possmayer, F. (1990). Pulmonary surfactant-associated protein A enhances the surface activity of lipid extract surfactant and reverses inhibition by blood proteins *in vitro*. *Biochemistry* 29, 8424-8429. 10.1021/bi00488a032 2252903

[JEB249280C5] Codd, J. R., Slocombe, N. C., Daniels, C. B., Wood, P. G. and Orgeig, S. (2000). Periodic fluctuations in the pulmonary surfactant system in Gould's wattled bat (*Chalinolobus gouldii*). *Physiol. Biochem. Zool.* 73, 605-612. 10.1086/317745 11073796

[JEB249280C6] Codd, J. R., Schürch, S., Daniels, C. B. and Orgeig, S. (2002). Torpor-associated fluctuations in surfactant activity in Gould's wattled bat. *Biochim. Biophys. Acta Mol. Cell Biol. Lipids* 1580, 57-66. 10.1016/S1388-1981(01)00185-8 11923100

[JEB249280C7] Duck-Chong, C. G. (1979). A rapid sensitive method for determining phospholipid phosphorus involving digestion with magnesium nitrate. *Lipids* 14, 492-497. 10.1007/BF02533467

[JEB249280C8] Goerke, J. and Clements, J. A. (1985). Alveolar surface tension and lung surfactant. In *Handbook of Physiology, the Respiratory System* (ed. S. R. Geiger), pp. 247-261. Bethesda: American Physiological Society.

[JEB249280C9] Gomez-Gil, L., Perez-Gil, J., Goormaghtigh, E., Gómez-Gil, L. and Pérez-Gil, J. (2009). Cholesterol modulates the exposure and orientation of pulmonary surfactant protein SP-C in model surfactant membranes. *Biochim. Biophys. Acta* 1788, 1907-1915. 10.1016/j.bbamem.2009.05.011 19464999

[JEB249280C10] Graham, E., McCaig, L., Shui-Kei Lau, G., Tejura, A., Cao, A., Zuo, Y. Y. and Veldhuizen, R. (2022). E-cigarette aerosol exposure of pulmonary surfactant impairs its surface tension reducing function. *PLoS One* 17, e0272475. 10.1371/journal.pone.0272475 36350850 PMC9645651

[JEB249280C11] Ikegami, M., Korfhagen, T. R., Whitsett, J. A., Bruno, M. D., Wert, S. E., Wada, K. and Jobe, A. H. (1998). Characteristics of surfactant from sp-a-deficient mice. *Am. J. Physiol.* 275, L247-L254. 10.1152/ajplung.1998.275.2.L247 9700084

[JEB249280C12] Lang, C. J., Postle, A. D., Orgeig, S., Possmayer, F., Bernhard, W., Panda, A. K., Jürgens, K. D., Milsom, W. K., Nag, K. and Daniels, C. B. (2005). Dipalmitoylphosphatidylcholine is not the major surfactant phospholipid species in all mammals. *Am. J. Physiol. Regul. Integr. Comp. Physiol.* 289, R1426-R1439. 10.1152/ajpregu.00496.2004 16037124

[JEB249280C13] Maruscak, A. A., Vockeroth, D. W., Girardi, B., Sheikh, T., Possmayer, F., Lewis, J. F. and Veldhuizen, R. A. W. (2008). Alterations to surfactant precede physiological deterioration during high tidal volume ventilation. *Am. J. Physiol. Lung Cell. Mol. Physiol.* 294, L974-L983. 10.1152/ajplung.00528.2007 18344412

[JEB249280C14] Pérez-Gil, J. (2008). Structure of pulmonary surfactant membranes and films: the role of proteins and lipid-protein interactions. *Biochim. Biophys. Acta* 1778, 1676-1695. 10.1016/j.bbamem.2008.05.003 18515069

[JEB249280C15] Possmayer, F., Zuo, Y. Y., Veldhuizen, R. A. W. and Petersen, N. O. (2023). Pulmonary surfactant: a mighty thin film. *Chem. Rev.* 123, 13209-13290. 10.1021/acs.chemrev.3c00146 37862151

[JEB249280C16] Puligandla, P. S., Gill, T., McCaig, L. A., Yao, L. J., Veldhuizen, R. A. W., Possmayer, F. and Lewis, J. F. (2000). Alveolar environment influences the metabolic and biophysical properties of exogenous surfactants. *J. Appl. Physiol.* 88, 1061-1071. 10.1152/jappl.2000.88.3.1061 10710404

[JEB249280C17] Schief, W. R., Antia, M., Discher, B. M., Hall, S. B. and Vogel, V. (2003). Liquid-crystalline collapse of pulmonary surfactant monolayers. *Biophys. J.* 84, 3792-3806. 10.1016/S0006-3495(03)75107-8 12770885 PMC1302961

[JEB249280C18] Slocombe, N. C., Codd, J. R., Wood, P. G., Orgeig, S. and Daniels, C. B. (2000). The effect of alterations in activity and body temperature on the pulmonary surfactant system in the lesser long-eared bat *Nyctophilus geoffroyi*. *J. Exp. Biol.* 203, 2429-2435. 10.1242/jeb.203.16.2429 10903157

[JEB249280C19] Sprenger, R. J. and Milsom, W. K. (2022). Ventilatory sensitivity to ambient CO_2_ at different hibernation temperatures in thirteen-lined ground Squirrels (*Ictidomys tridecemlineatus*). *Physiol. Biochem. Zool.* 95, 288-301. 10.1086/720158 35588474

[JEB249280C20] Staples, J. F. (2016). Metabolic flexibility: hibernation, torpor, and estivation. *Compr Physiol.* 6, 737-771. 10.1002/cphy.c140064 27065167

[JEB249280C21] Suri, L. N. M., McCaig, L., Picardi, M. V., Ospina, O. L., Veldhuizen, R. A. W., Staples, J. F., Possmayer, F., Yao, L.-J., Perez-Gil, J. and Orgeig, S. (2012). Adaptation to low body temperature influences pulmonary surfactant composition thereby increasing fluidity while maintaining appropriately ordered membrane structure and surface activity. *Biochim. Biophys. Acta* 1818, 1581-1589. 10.1016/j.bbamem.2012.02.021 22387458

[JEB249280C22] Suri, L. N. M., Cruz, A., Veldhuizen, R. A. W., Staples, J. F., Possmayer, F., Orgeig, S. and Perez-Gil, J. (2013). Adaptations to hibernation in lung surfactant composition of 13-lined ground squirrels influence surfactant lipid phase segregation properties. *Biochim. Biophys. Acta Biomembr.* 1828, 1707-1714. 10.1016/j.bbamem.2013.03.005 23506681

[JEB249280C23] Valle, R. P., Wu, T. and Zuo, Y. Y. (2015). Biophysical influence of airborne carbon nanomaterials on natural pulmonary surfactant. *ACS Nano* 9, 5413-5421. 10.1021/acsnano.5b01181 25929264 PMC4856476

[JEB249280C24] Whitsett, J. A. and Weaver, T. E. (2002). Hydrophobic surfactant proteins in lung function and disease. *N. Engl. J. Med.* 347, 2141-2148. 10.1056/NEJMra022387 12501227

[JEB249280C25] Zhang, H., Wang, Y. E., Neal, C. R. and Zuo, Y. Y. (2012). Differential effects of cholesterol and budesonide on biophysical properties of clinical surfactant. *Pediatr. Res.* 71, 316-323. 10.1038/pr.2011.78 22391630 PMC3338335

[JEB249280C26] Zuo, Y. Y., Ding, M., Li, D. and Neumann, A. W. (2004). Further development of Axisymmetric Drop Shape Analysis-captive bubble for pulmonary surfactant related studies. *Biochim. Biophys. Acta* 1675, 12-20. 10.1016/j.bbagen.2004.08.003 15535963

[JEB249280C27] Zuo, Y. Y., Gitiafroz, R., Acosta, E., Policova, Z., Cox, P. N., Hair, M. L. and Neumann, A. W. (2005). Effect of humidity on the adsorption kinetics of lung surfactant at air-water interfaces. *Langmuir* 21, 10593-10601. 10.1021/la0517078 16262325

[JEB249280C28] Zuo, Y. Y., Veldhuizen, R. A. W., Neumann, A. W., Petersen, N. O. and Possmayer, F. (2008). Current perspectives in pulmonary surfactant - Inhibition, enhancement and evaluation. *Biochim. Biophys. Acta* 1778, 1947-1977. 10.1016/j.bbamem.2008.03.021 18433715

